# A computational method for studying the relation between alternative splicing and DNA methylation

**DOI:** 10.1093/nar/gkv906

**Published:** 2015-09-13

**Authors:** Zejun Zheng, Xiaona Wei, Andreas Hildebrandt, Bertil Schmidt

**Affiliations:** 1 Bioinformatics Institute, Singapore 138671, Singapore; 2 Institute of Bioengineering and Nanotechnology, Singapore 138669, Singapore; 3 Institut für Informatik, Johannes Gutenberg Universität Mainz, 55099 Mainz, Germany

## Abstract

Alternative splicing is an important mechanism in eukaryotes that expands the transcriptome and proteome significantly. It plays an important role in a number of biological processes. Understanding its regulation is hence an important challenge. Recently, increasing evidence has been collected that supports an involvement of intragenic DNA methylation in the regulation of alternative splicing. The exact mechanisms of regulation, however, are largely unknown, and speculated to be complex: different methylation profiles might exist, each of which could be associated with a different regulation mechanism. We present a computational technique that is able to determine such stable methylation patterns and allows to correlate these patterns with inclusion propensity of exons. Pattern detection is based on dynamic time warping (DTW) of methylation profiles, a sophisticated similarity measure for signals that can be non-trivially transformed. We design a flexible self-organizing map approach to pattern grouping. Exemplary application on available data sets indicates that stable patterns which correlate non-trivially with exon inclusion do indeed exist. To improve the reliability of these predictions, further studies on larger data sets will be required. We have thus taken great care that our software runs efficiently on modern hardware, so that it can support future studies on large-scale data sets.

## INTRODUCTION

Alternative splicing (AS) significantly expands the transcriptome and proteome diversity in higher eukaryotic cells (1). About 95% of human precursor messenger (pre-mRNA) contains at least one exon that is processed to yield multiple mRNA isoforms (2). A recent survey of the *Arabidopsis* transcriptome has revealed that under normal growth conditions about 61% of multi-exonic genes are alternatively spliced (3). Furthermore, aberrant splicing can be associated with a wide spectrum of diseases (4).

Intragenic *DNA methylation* is an emerging candidate of a mechanism for exon splicing regulation. A genome-wide study (5) has reported that human exons are more highly methylated than introns and methylation differences are stronger at the exon–intron boundaries. Thus, DNA methylation could play a role in the control of transcript splicing.

A subsequent epigenetic survey of distribution factors performed on human cell lines (6) has shown differential association patterns between DNA methylation and various AS sites. This suggests that DNA methylation is likely to play an important role in regulating AS. The genome-wide association between DNA methylation and AS has also been observed in *Apis mellifera* (7). The observed AS occurs in significantly higher frequency in methylated genes as compared to un-methylated genes. Furthermore, methylation levels are higher in included exons than in excluded exons. The enhancement of DNA methylation in exon inclusion has been found to be related to MeCP2 mediated transcription repression (8). DNA methylation has also been reported to inhibit the inclusion enhancement mediated by CTCF (9). These observations indicate that the regulation of DNA methylation on AS is complex and involves several factors and diverse mechanisms. Therefore, AS exons with different methylation regulation mechanisms could have different DNA methylation profiles. For example, a recent study (10) hinted that exons with different GC contents could be regulated by different splicing mechanisms, which could be related to different DNA methylation profiles.

In this paper we present a computational approach that can categorize the relation between AS exons and DNA methylation using next-generation sequencing (NGS) data. Specifically, our approach addresses two questions that are of high importance to genomic research:

1. Does AS exon DNA methylation form stable profile patterns that are independent of cell types?
2. How are DNA methylation and AS exon inclusion linked with various other properties of exon splicing sites taking different methylation profile groupings into consideration?

Genome-wide categorization of DNA methylation (or other epigenetic track profiles) is made challenging by the diversity of epigenetic profiles. For example, DNA methylation is affected by various factors such as sequence composition transcriptional factor binding (11) and nucleosome positioning (12). Thus, epigenetic profiles of regions with similar function can be globally similar but may have local differences. These differences could be caused by transformations such as shrinking, stretching or shifting, leading to unequal exon and intron lengths. Our developed epigenetic profile typing algorithm therefore adopts the dynamic time warping (DTW) method (13) for the measurement of similarity between epigenetic profiles generated from NGS data. DTW is a symmetric distance measure that does not obey the triangle inequality (14). This property makes it difficult to integrate DTW into clustering algorithms that rely on an averaging method. Thus, we have designed an elastic self-organizing map (SOM)-based (15) approach for the typing of epigenetic profiles with varying lengths, which features an effective DTW-based averaging scheme for cluster consensus representation. We show that this method can categorize skipping event (SE) exons and retained intron (RI) events into stable sub-groups independent of the cell and tissue types. This suggests common methylation mechanisms on the regulation of AS events among different (human) tissues.

## MATERIALS AND METHODS

### Data preparation

We have tested our algorithm with eight sets of matched methylation/RNA-seq data from different human tissues obtained from the NIH Roadmap Epigenomics Mapping Consortium (http://www.roadmapepigenomics.org/). We have selected those data sets for which both RNA-seq and bisulfite-sequencing have been generated from the same sample to minimize sample variation effects. Gene Expression Omnibus (GEO) accession numbers of the data sets are listed in Table 1.

**Table 1.** GEO accession numbers of the methylation and transcription sequencing data sets used in this study

Tissues/cell lines	Methylation (BS-Seq)	Transcription (RNA-seq)
Adipose	GSM1120331	GSM1010958
Esophagus	GSM983649	GSM1010956
HUES64	GSM1112840	GSM1112834
Lung	GSM983647	GSM1010946
MobCD34	GSM916052	GSM909310
Ovary	GSM1120323	GSM1010948
Pancreas	GSM983651	GSM1010966
Thymus	GSM1120322	GSM1010944

We mainly focus on SE events for the evaluation of our method since SEs are the most abundant AS event type. Nevertheless, we have also tested the RI event type to evaluate our method in a rarer AS case (16). For the typing of SE and RI events based on methylation profiles, we have expanded +200 bp and −200 bp from the up- and down-stream borders for each AS event (SE or RI). However, the uniform expansion of 200 bp to each side of a border can be problematic, because the expansion can cross the neighboring exon/intron border. In such cases, we stop the expansion early, leading to regions of potentially unequal size.

In order to determine the inclusion levels of the SE exons, RNA-Seq reads are aligned to the human hg19 reference genome with the splice junction mapper TopHat v.1.3.2 (17). Only uniquely aligned reads are kept. The aligned reads are indexed using SAMtools v.0.1.19 (18) and then AS levels are quantitatively estimated using MISO v.0.4.9 (19). The fragments per kilobase per million mapped reads (FPKM) for transcripts are also calculated with cufflinks v2.2.1 (20). For DNA methylation information, we adopt the author provided data on GEO. For the subsequent shape-based epigenetic track typing, we have digitized methylation levels throughout the whole genome in 20 bp resolution and perform a quantile normalization across all samples.

### Dynamic time warping of epigenetic profiles

We use the concept of Dynamic Time Warping (DTW) (14) for measuring the similarity between two methylation profiles. Consider two real-valued sequences *S*_1_ = (*x*_0_,…,*x_n_*_−1_) and *S*_2_ = (*y*_0_,…,*y_m_*_−1_) of length *n* and *m* respectively, where *x_i_* (*y_j_*) represent the read density of the *i*^th^ (*j*^th^) location on *S*_1_ (*S*_2_) in our application. Let *I*: = dom(*S*_1_) and *J*: = dom(*S*_2_) be the index sets of *S*_1_ and *S*_2_. The sequence of tuples γ: = ((*i_l_*,*j_l_*)∈*I*×*J*)*_l_* is called a monotone, continuous and bounded warping path if and only if $\min (i_{l + 1} - i_l ,j_{l + 1} - j_l ) \ge 0$ and $\max (i_{l + 1} - i_l ,j_{l + 1} - j_l ) = 1$, $\forall l \in \left\{ {1,...,\left| \gamma \right| - 2} \right\}$, where (*i*_0_,*j*_0_) = (0,0) and $(i_{\left| \gamma \right| - 1} ,j_{\left| \gamma \right| - 1} ) = (n - 1,\;m - 1).$

The fundamental properties of DTW are derived directly from this definition.

1. *Continuity*. Consecutive nodes in γ must be reached by horizontal, vertical or diagonal steps of length 1. Hence, DTW matches every index of *S*_1_ and *S*_2_ without any gaps.
2. *Monotonicity*. Each segment of the warping path γ has to increment at least one index of *S*_1_ or *S*_2_. As a result, DTW is not allowed to map an index tuple (*i_l_, j_l_*) several times.
3. *Bounding*. The warping path starts at the first index of *S*_1_ and *S*_2_. Analogously, ends at the last index. Therefore, DTW is a global alignment of two real-valued sequences (or time series).

**Figure 1.**
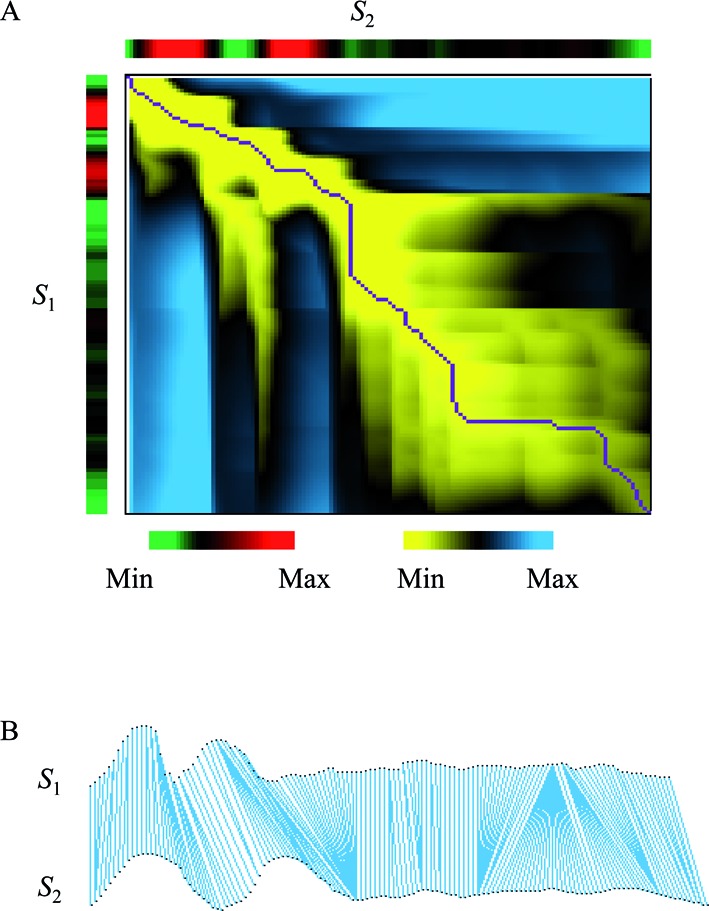
DTW of two methylation profiles (*S*_1_ and *S*_2_). (**A**) Dynamic programing (DP) matrix. The optimal warping path is marked in purple. Signal insentities are color coded using a green (low) to red (high) scale. The cumulative cost for each element in the DP matrix is marked from yellow (low) to blue (high). (**B**) The alignment corresponding to the optimal warping path.

The unification of all warping paths is a directed and acyclic graph (DAG). Non-negative weights are assigned to all incoming edges of a node (*i_l_, j_l_*) by $w(i_l ,j_l ): = (x_{i_l } - y_{j_l } )^2$. During the further procedure, DTW calculates the optimal warping path with minimal sum of weights. Figure 1 illustrates an example of an optimal warping path during DTW relaxation of two epigenetic profile signals.

Let Γ be the set of all monotone, continuous and bounded warping paths. The *optimal warping path* $\hat \gamma$ and its *associated measure* $\hat d$ with respect to a given weighting function $w:I \times J \to \Re _0^ +$ are defined as: $\hat \gamma : = \mathop {\arg \min }\limits_{\gamma \in \Gamma } \sum\limits_{(i_l ,j_l ) \in \gamma } {w(i_l ,j_l )}$ and $\hat d: = \mathop {\min }\limits_{\gamma \in \Gamma } \sum\limits_{(i_l ,j_l ) \in \gamma } {w(i_l ,j_l )}$.

This optimization problem is equivalent to the calculation of a shortest path within a DAG. The relaxation of a cell (*i*,*j*) of the penalty matrix *M* can be written recursively as *M*[*i*,*j*] = *w*(*i*,*j*) + min{*M*[*i*−1,*j*], *M*[*i*,*j*−1], *M*[*i*−1,*j*−1]}. An implementation is usually achieved by dynamic programming with *O*(*n*×*m*) runtime.

The described relaxation scheme for DTW determines the optimal warping path on the whole graph. Empirical studies (21) suggest that DTW's quality (in means of kNN-classification error) can be increased for time series of approximately equal length by restricting warping paths to the neighborhood of the main diagonal. The Sakoe-Chiba band excludes nodes on the upper right and lower left region of the penalty matrix. As a result, pathological alignments are excluded which may increase classification quality. Thus, we have also adapted the banded DTW approach in our implementation. Optimal warping path distances are further normalized by the path length.

### DTW-based self-organizing map

The self-organization map (SOM) (22) is a neural network model that maps high-dimensional input data onto a topologically organized grid of neurons. Neurons are represented by weighted vector functions (*W*). During the training procedure, each input data item (*S*) is assigned to the best matching unit (BMU). Weighted vectors close to the BMU are incrementally adjusted toward the input data, where the adjustment force decreases with the grid distance to the BMU. The impact of the adjustment is iteratively reduced with each learning epoch (*t*). Since the general SOM method is well-known, we will focus on our flexible weighted vector adjustment strategy. Updating the weight vector relies on some type of averaging operation. Defining this averaging operation for our application scenario is a non-trivial task because it has to be consistent with the ability of DTW to realign sequences over time (23).

**Figure 2.**
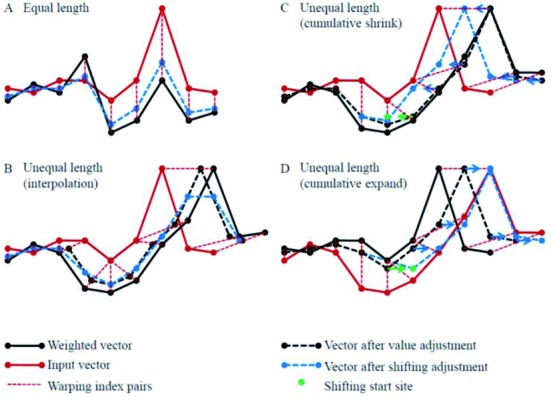
Strategies for the adjustment of the weighted vector in the SOM learning process of methylation profiles. (**A**) Optimal warping path is along the main diagonal of the DP matrix. In this case, no length adjustment is involved, the adjusted weighted vector (blue dashed line) is simply the weighted average of the two. (**B**) The adjusted weighted vector (black dashed line) has non-integer indices (black dots). Element values at integer positions are generated by interpolation (blue dashed line). Interpolation can severely twist the adjustments. (**C**) and (**D**) The FSOM strategy accumulates the index adjustment for each element of the weighted vector avoiding adjustment of indices for each iteration. The adjustment is only triggered when the accumulator exceeds an integer-valued threshold. In this case, if the length of the index (dot with arrow) is to be reduced ((C), shrinking) or extended ((D), expansion), the index of the relevant element is reduced by an integer length (round to integer, indicated by green dots).

Firstly, for a given methylation profile *S* = (*x*_0_,…,*x_n_*_−1_) the BMU (*U*) is determined and the relevant neighboring neurons within range *r* = *h*(*U*,*t*) are adjusted. For a neuron represented by the weighted vector *W* = (*y*_0_,…,*y_m_*_−1_) with distance *r*≤*R* to the BMU we compute the optimal warping path *P* = ((*i_l_*,*j_l_*)∈dom(*S*)×dom(*W*))*_l_* between *S* and *W*. For the simple case (illustrated in Figure 2A) when *n* = *m* and the warping path is along the main diagonal of the DP matrix, the weighted vector can be adjusted by simply computing $y\prime _{j_l } = y_{j_l } + \alpha (t,r)(x_{i_l } - y_{j_l } )$ for each warping path index pair (*i_l_*,*j_l_*) where α(*t*,*r*) is the learning force function depending on the radius *r* and the learning epoch *t*.

In the general case, shrinking and expansion between *W* and *S* with respect to *P* must also be considered. Figure 2 illustrates four possible scenarios. For two consecutive elements in *P*, (*i_k_*,*j_k_*) and (*i_k_*_+1_,*j_k_*_+1_), we define three cases:

1. *Shrinking*: *i_k_*_+1_ > *i_k_* and *j_k_*_+1_ = *j_k_*
2. *Expansion*: *i_k_*_+1_ = *i_k_* and *j_k_*_+1_ > *j_k_*
3. *Diagonal*: *i_k_*_+1_ > *i_k_* and *j_k_*_+1_ > *j_k_*

A possible solution to update the indices of *W* for Cases (1) and (2) could be designed by using interpolation of weighted vector values at integer index locations. Unfortunately, this approach would allow the values of the weighted vector to become significantly twisted (see Figure 2B). Thus, a more suitable approach would be to restrict the interpolation within the shrinking and expansion sites and only make integer shifts of the rest (see Figure 2C and D). Thus, we restrict the use of interpolation by using an accumulative strategy for index adjustment (called FSOM (Flexible weighted vector adjustment based SOM)). FSOM accumulates adjustments for each of the elements in the weighted vector. Index adjustment is only triggered when the accumulated adjustment value reaches a threshold. In this case the indices at the relevant location (e.g. *j_k_*) are shifted by one and the accumulator is reset to zero. The same unit of shifting is also applied to all indices larger than *j_k_* but their accumulator values keep unchanged.

The limited value adjustment caused by index adjustment occurs when an index shifting is triggered. For the expansion case, new values created by interpolation occupy the expanded indices (see Figure 2D). For the shrinking case, index overlapping is caused by shifting, where we average the values of overlapping elements (see Figure 2C).

### Light-weight algorithm for determining the number of clusters

Before applying FSOM it is necessary to determine the number of subtypes to be trained by FSOM. To be biologically meaningful, this number should reflect the natural data distribution. The consensus clustering method (24) determines the number of clusters that best fit the data based on the concept of consensus matrices. A consensus matrix stores for each pair of objects the proportion of clustering runs in which this pair is clustered together. The consensus matrix of a given cluster size can be obtained by averaging over the connectivity matrices of every perturbed data set (sub-sample). Thus, clustering has to be performed a large number of times (for each sub-sample and for each tested cluster size (or number of groups)). Since this approach is highly time-consuming for our computationally expensive DTW-based SOM method, we have designed a faster approach to address this problem.

The consensus clustering approach scans a range of group numbers *k* with 2 ≤ *k* ≤ *K*. For each value of *k* the algorithm performs a partitioning calculation using *m* sub-samples and then calculates a consensus matrix from co-assignments. The optimal value of *k* shows maximal intra-group connectivity/similarity and minimal inter-group connectivity. The quality of a partitioning is measured by the area under the empirical cumulative distribution function (CDF) which is calculated from the consensus matrix (24).

Our approach computes the consensus matrix in a more efficient way. We consider the *k*-range of 4 ≤ *k* ≤ *K* (as the minimal number of groups for SOM is four). To compute the co-assignment of data items for several values of *k*, we perform the partitioning calculation only once with a grid of *M* groups (neurons) making it computationally more efficient than the original consensus clustering method. Our approach consists of the following three steps.

1. Compute the partitioning with a grid setting consisting of *M* groups. Record the distance of data items to all *mediods* (i.e. the weighted vector for SOM). For each data item, we rank the mediods by similarity.
2. For each *k*, sample *k* mediods and assign all the data items to these *k* mediods. We can assign each data item to the nearest mediod according to the ranking of mediods recorded in Step (1) without the need for repeatedly computing the distances. The assignment is repeated *m* times to compute the co-assignment of the data item pairs. As $\left( {\begin{array}{*{20}c} M \\ K \\\end{array}} \right) >m$, we can ensure that it is possible to sample *m* sets of mediods for all *k* ≤ *K*.
3. Compute the area under CDF for all 4 ≤ *k* ≤ *K* and determine the optimal value k*.

### Cluster validation

Normalized mutual information (NMI) is used as the criterion for evaluating the agreement between two groupings produced by different SOM runs. The NMI measure returns a score in the range [0,1]. A random labeling in which the two groupings are very different results in an NMI score close to 0.0 while a perfect agreement has a score of 1.0.

### Unsupervised random forest learning

In order to determine whether our DTW-based FSOM method has additional value beyond a simple feature-based grouping approach, we train an Unsupervised random forest (URF) using global properties of SE events, such as average methylation level, GC-/CpG-ratio and upstream intron length of I/E and E/I boundaries. URFs are a powerful approach to perform unsupervised learning tasks (25). Hence, if our more complex DTW-based approach has additional value, it should produce more meaningful clusters than a properly trained URF. The URF model used in our tests has been trained with the R package *randomForest* using the recommended parameters (26).

### Statistical tests

For investigating the association between produced AS clusters and various biological properties we test each property within each group against the background (which we define as the entire AS set for each of the tissues) using the Mann–Whitney U-test. The tested biological properties include the AS level score Ψ, exon/intron lengths, distance to TSS, GC and CpG ratios, and expression levels (FPKM) of host genes. For the GC and CpG ratio test on the intron–exon (I/E) and exon–intron (E/I) border regions, we divide the regions into 20 bp bins and then test each of them individually against the background set. Clusters for which more than 80% of those bins show the same significant trends are marked as significant. For testing of motif enrichment/depletion in each of the clusters we use a hypergeometric test. We set the false discovery rate (FDR) to level 0.05. All statistical tests are performed with the R package (http://www.r-project.org/).

## RESULTS

### FSOM typing of Adipose SE based on methylation profiles

**Figure 3.**
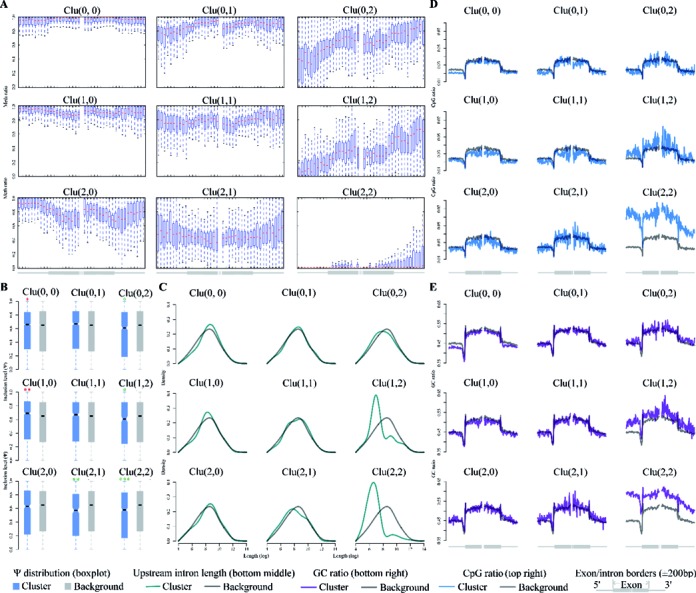
FSOM typing of Adipose tissue SE events based on methylation profiles. (**A**) FSOM typing of Adipose tissue SE events based on methylation levels on an ±200 bp interval for I/E and E/I borders. Methylation profiles for each 20 bp bin of the same cluster are presented by a boxplot. Median methylation levels are shown as red dashed lines. (**B**) Inclusion levels (in terms of Ψ score) for each of the clusters (blue) compared to the overall inclusion background (gray). The inclusion levels vary from significantly high (red asterisk) to significantly low (green asterisk) and follow the same trends as the methylation levels in (A). (**C**) Association between SE clusters and up-stream intron lengths with the entire SE events set as background. (**D**) Average CpG level of the clusters (blue) along with the background (gray). CpG ratios have a negative association with methylation profile changes. (**E**) Average GC level for clusters (purple) compared with background (gray). The GC ratio also has negative association with methylation profile changes.

We have tested our FSOM method on data from eight tissues/cell lines (Adipose, Esophagus, HUES64, Lung, MobCD34, Ovary, Pancreas and Thymus; see also Table 1). The number of detected SE events ranges from 15 237 to 30 236. We now describe the results obtained on the Adipose data in detail (Figure 3), while the FSOM clustering results for the other tissue types are given in the supplement (Supplementary Figures S1–S7).

First, we employ the NMI criteria (see Materials and Methods) for examining the stability clusters with respect to the FSOM grid size. The resultant NMI scores are >0.82 for all the tests on the Adipose data with topological grid settings ranging from 2 × 2 to 3 × 6. We determine the optimal grid setting with our light-weight algorithm (see Materials and Methods) resulting in a 3 × 3 grid setting. Figure 3A shows the resulting nine clusters after FSOM typing based on the combined methylation profiles on the proximal ±200 bp of the E/I and I/E borders.

As methylation levels can be associated with multiple factors, e.g. CpG architecture and exon/intron length, the shape of the methylation profiles can help to find SEs of the same category. We therefore test multiple factors for association with methylation profiles on the SE borders including CpG ratio, GC ratio, residing gene expression, SE exon length, up-/down-stream intron length, distance to TSS and inclusion level (Ψ score).

The FSOM method returns clusters that show a gradient from high-to-low methylation levels along rows and columns of the 3 × 3 grid (see Figure 3A). For most of these clusters, the methylation profile is condensed to a narrow band that follows a certain shape pattern characterizing the cluster. For example, the least methylated cluster (Figure 3A, Clu(2,2)) shows significantly lower methylation levels on the upstream intron side compared to the downstream border region suggesting that the low methylation on the I/E border could be important for splicing regulation of this cluster. The clustering results for the other seven tissues/cell lines show similar trends (see Supplementary Figures S1–S7), whereby the enumeration of the clusters may vary; e.g. Clu(0,0) and Clu(2,2) in Adipose correspond to Clu(2,0) and Clu(0,2) in Esophagus.

Furthermore, Figure 3B to E show the other tested properties per cluster. Even though these properties have not been used by the clustering procedure, each property show similar trends along the dimensions of the clustering matrix.

Next, we have tested whether SE methylation profiles are affected by the lengths of exon and flanking introns. We have hence tested the association between the SE clusters and the lengths of exons and up-/down-stream introns with the entire SE event set as background (see Figure 3C). The largest correlation was found for the upstream intron length, which changes from significant longer than background (Mann–Whitney U-test, *P* < 0.05, FDR 0.05) to significant shorter than the background. A pronounced association is that the low methylation cluster Clu(2,2) shows also significant association with upstream flanking intron length (Figure 3C) which further suggests the importance of the upstream intron for the regulation of the SE events contained in this cluster.

GC and CpG architecture are known to have important impact on SE E/I and I/E border methylation patterns (10). We further show that the GC (Figure 3E) and CpG (Figure3D) ratio has a negative association with the methylation profile change. Furthermore, our data indicate that clusters with higher overall methylation levels (e.g. Clu(0,0)) show differential CpG and GC ratio on exon and intron side regions while clusters with lower methylation levels (e.g. Clu(1,2) and Clu(2,2)) show similar GC and CpG ratio on exon and intron side regions.

Along with the methylation profile changing, the inclusion level shows positive correlation with overall SE methylation levels (see Figure 3B). However, this does not necessarily mean that, for a specific SE event, when the inclusion level changes, the methylation profile will shift from one cluster to another. Actually, as it will be discussed in the next section, by testing all the eight tissues, the grouping of AS events is relatively conserved. Thus, the different splicing regulation mechanism may encode the basal methylation level of these groups.

### Conserved SE methylation profile grouping across tissues/cell lines

We have tested our DTW-based FSOM method on the eight tissues/cell lines listed in Table 1 to investigate whether the calculated methylation profile groups share common AS regulation mechanisms across different tissues/cell lines.

**Figure 4.**
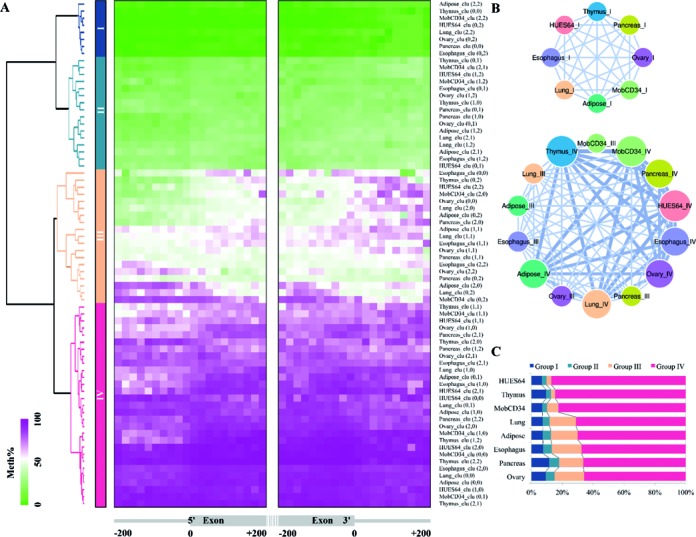
Stability of FSOM clustering across eight tissues/cell lines. (**A**) Complete linkage hierarchical clustering of the 8 × 9 = 72 SE methylation clusters. Each row in the heatmap presents the methylation prototypes for one cluster with methylation levels coded on a green (low) to purple (high) scale. Four groups (Group I–IV) of SE methylation clusters are defined by cutting the dendrogram at the second level. (**B**) Analysis of the shared membership between Group I (low methylation) and Groups III and IV (high methylation). The graph illustrates the pairwise membership sharing by weighted edges (percentage of total common SEs, edges with <5% of total common SEs are omitted). Groups I and IV have less than 5% members in common among all the tested tissues. (**C**) Membership ratio map. From the ratio map we can observe that Group IV represents the most common SE regulation mechanism and that the highest relative ratio variation is <20%.

To combine the FSOM results (3 × 3 grid size) from each tissue, we have computed a complete linkage hierarchical clustering of the resulting 8 × 9 = 72 methylation clusters (see Figure 4A). The hierarchical clustering uses DTW computed on the median methylation levels of each cluster as distance measure. We then cut the resulting dendrogram at the second level to produce four groupings (Groups I–IV). The four groups show an increase in overall methylation levels from Group I to IV (Figure 4A). Note that each group contains at least one representative from each tissue/cell line. Furthermore, the highest relative ratio variation is <20% (see Figure 4C). From the ratio map in Figure 4C we can further observe that Group IV represents the most common SE regulation mechanism.

Membership of SE events is relatively stable in the sense that shared membership between Group I (low methylation) and Group IV (high methylation) is rare (<5% of commonly covered SE event, *n* = 6125). Shared membership between Group I and between Group III and IV is shown in Figure 4B. This observation suggests that the majority of SE events has a fixed methylation pattern that is resistant to dramatic changes of methylation among different tissues.

Furthermore, Supplementary Figure S8 shows the corresponding hierarchical clustering for the RI methylation clusters which show similar trends.

### Association of SE and RI methylation and various biological properties

**Figure 5.**
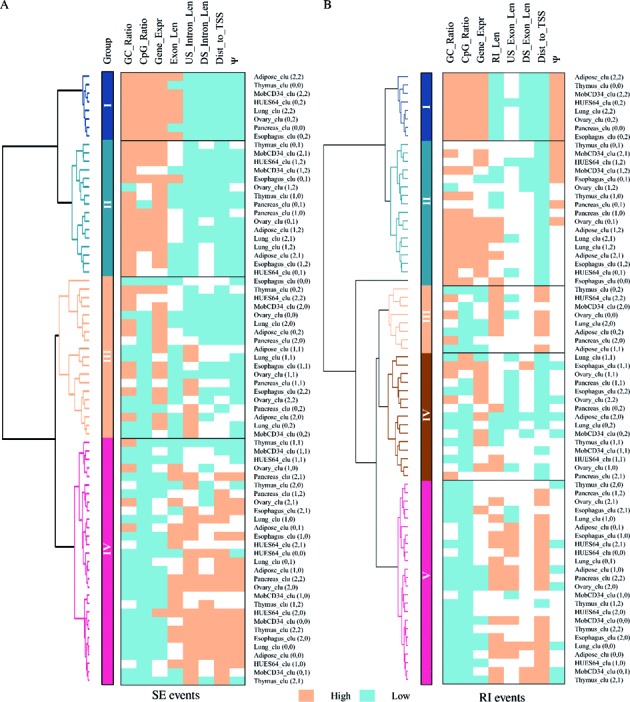
Correlation between several biological properties (CpG ratio, GC ratio, host gene expression, SE exon length, up-/down-stream intron length, distance to TSS, Ψ score) and produced SE (**A**) and RI (**B**) methylation groupings. Each test compares to the background level and is marked as significant (*P* < 0.05, FDR 0.05) in either blue (high) or orange (low), or as insignificant (in white).

To further validate the performance of our typing method we have investigated the correlation between several biological properties and the produced SE and RI methylation groupings. Our results for SE methylation are summarized in Figure 5A. We can observe that SE groups I and II show higher levels of GC and CpG ratios throughout the border regions, while Group IV shows lower levels.

Gene expression levels are investigated as another factor that could affect the DNA methylation pattern modeling. FPKM levels of host genes show indeed an association with the methylation grouping. This suggests that the methylation modeling of SE borders reflects both, local exon splicing regulation and the entire gene expression status, which tunes the methylation profiles on SE borders. The relative distance of the SE border to the TSS site is a further property that we consider to have impact on SE methylation regulation since methylation's role in regulating transcription is well known (5). Group IV shows shorter distance than the other groups (U-test).

In summary, all eight investigated associations show a degree of conservation across all eight tissues/cell lines (see Figure 5A). Similar conservation can also be observed in RI methylation typing across the eight tissues/cell lines (see Figure 5B), although the smaller data size leads to less sensitivity of the statistical tests.

### Splicing motif binding regulated by methylation profiles

**Figure 6.**
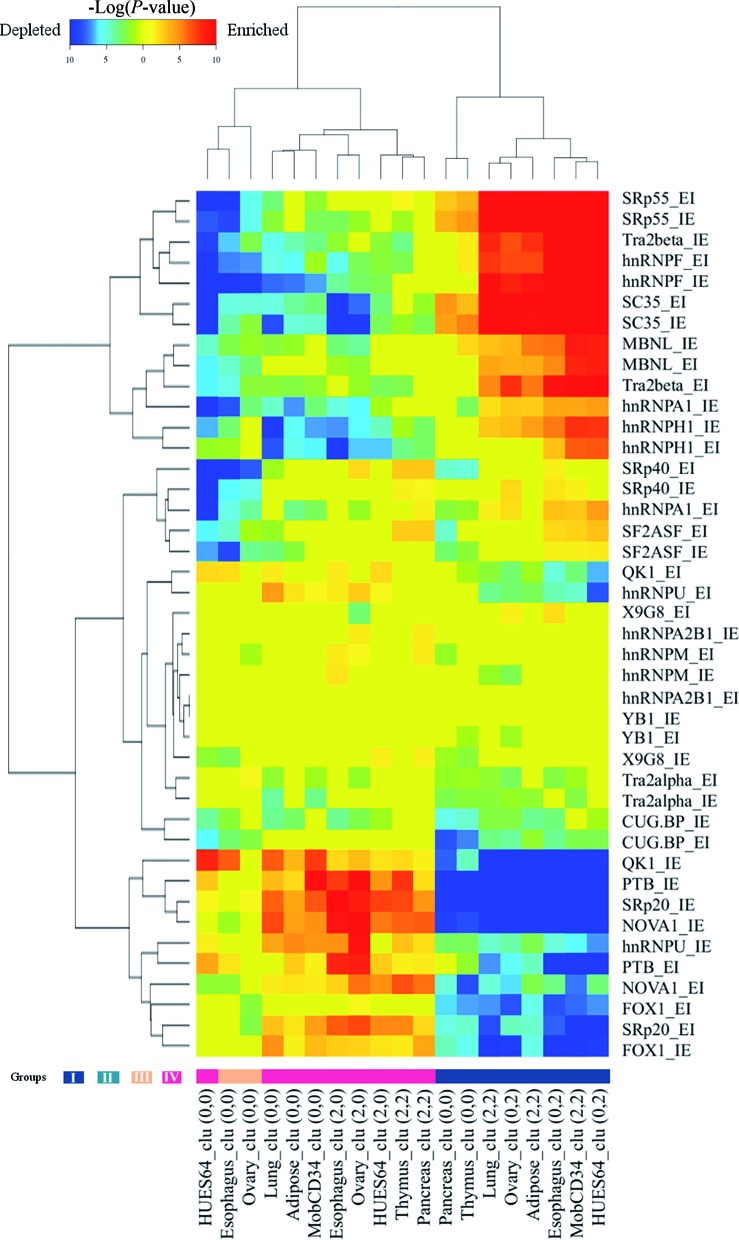
Splicing motif enrichment and distribution among different SE clusters. Motif enrichment and depletion are tested for all of the clusters (hypergeometric test). Only clusters with significant (*P* < 0.05, FDR 0.05) motif enrichment and depletion are shown (see Figures S10 and S11 for all clusters). Motif enrichment (warm color) and depletion (cold color) are shown on a −log10(*P*) color scale. Methylation groupings are shown at the bottom. Both I/E and E/I border regions are tested.

Splicing motif binding is a crucial step for regulation of exon splicing and may also suggest differential splicing regulation mechanisms. To further characterize the methylation typing of SE groups, we have tested the enrichment/depletion of splicing motifs on the SE border regions for all the clusters across the eight tissues/cell lines. We would expect that the motifs show a conserved enrichment/depletion pattern for the same categories of SEs across different tissues/cell lines. We can observe two distinct groups (I versus IV and III) of SEs that have reversed enrichment/depletion patterns involving 13 known splicing motifs (see Figure 6 and Supplementary Figure S9). Splicing motifs for QK1, PTB, SRp20, NOVA1, hnRNPU, PTB and FOX1 are enriched in the highly methylated groups (Group III and Group IV) while depleted from the low methylated SE group (Group I). On the other hand, motifs for SRp55, Tra2beta, hnRNPF, SC35, MBNL and hnRNPA1 are enriched in Group I while depleted from Group III and Group IV. The enrichment/depletion pattern is highly consistent across different tissues/cell lines.

The distinct motif distribution of different SE groups further supports the existence of different AS regulation mechanisms that are commonly performed in different tissue types. These mechanisms probably affect a fixed spectrum of SE exons suggested by the highly conserved membership of the major methylation typing groups.

### Additional value beyond a simple feature-based grouping approach

We have previously observed that various biological properties correlate well with the discovered methylation groupings. Thus, we have further tested whether meaningful groupings could also be recovered from these properties without the use of our DTW-based FSOM approach.

**Figure 7.**
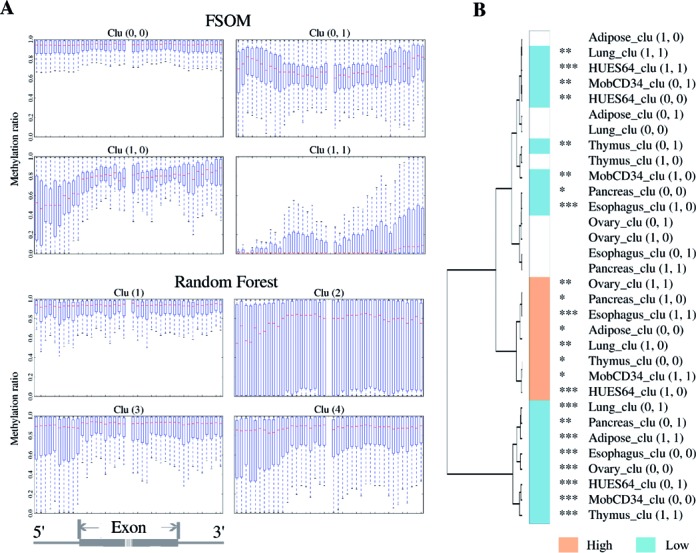
(**A**) Comparison of our FSOM method with a 2 × 2 grid size and an URF learned from global properties with the number of clusters set to four for Adipose SE events. (**B**) FSOM clustering of SEs with a 2 × 2 grid setting yields stable typing of methylation groups. The counterpart for the two distinct groups (Clu(0,1) and Clu(1,1)) of Adipose SEs can be found in all the eight tissues. The significance of SE inclusion levels is also indicated for each cluster.

We have thus trained an unsupervised random forest (URF) (see Materials and Methods) on such properties, namely average methylation level, GC-/CpG-ratio, and upstream intron length of I/E and E/I boundaries. For both methods (FSOM and URF), we have set the number of clusters to four, corresponding to the four major superclusters we found. Figure 7, Supplementary Figures S10 and S11 display the clusters resulting from our approach to those from URF.

The results show that the URF learning model fails to find common methylation profile patterns of SE groups. Therefore, it is also not able to detect stable association patterns between methylation profiles and SE inclusion. For example in HUES64, MobCD34 and thymus, the URF generates two groups that show high methylation profiles. However, the association between these methylation profiles and the inclusion levels shows no recognizable structure. Instead the URF clusters with high methylation levels are associated with both high-and low-inclusion levels (see Supplementary Figures S10 and S11), and hence describe tissue-specific effects rather than general AS regulation mechanisms.

In contrast, our method considers shape information by using a flexible DTW-based approach instead of simple global features. The fact that our method is able to outperform methods based on global properties implies that there is valuable information in the shape of the methylation profiles of AS events. A possible explanation of the influence of the methylation shape on AS regulation is the existence of symphonized mechanisms which leave their ‘footprints’ on the methylation profile. Hence, knowledge about the shape may be crucial to decipher the original AS regulation groups.

Our experimental results using an FSOM grid of size 2 × 2 (see Figure 7B) and of size 3 × 3 (see Figure 4A) also indicate that groupings across tissues/cell lines are somewhat stable with respect to the number of utilized cluster prototypes.

## DISCUSSION

In this work, we have presented a computational technique for the study of stable methylation patterns which is a crucial piece of the puzzle in the attempt to understand the influence of DNA methylation on the regulation of AS.

The idea behind searching for patterns is as follows: an effect of methylation on AS can either be a result of bulk properties (such as total GC content), of higher-order effects or of a combination of the two. For instance, a certain AS event might require a certain total GC content, but also a certain shape of the methylation pattern. Studying the effect of bulk properties is simple: we can just compute them for test examples and see how they correlate with the outcome. But to understand the effect of higher-order patterns, we first need a method to robustly and efficiently detect these from the data. Then, we can cluster data into instances with similar methylation profile and study whether the members of the resulting clusters tend to correlate in similar ways with the outcome.

Detecting such patterns is a non-trivial challenge: evolutionary changes can stretch, shrink or shift parts of the pattern. Hence, a simple correlation of methylation profiles as a function of sequence position is likely to be too simplistic as a measure of methylation similarity. Instead, we propose the use of the DTW algorithm known from time series analysis, which can be made invariant with respect to the above transformations. However, integrating DTW into the clustering schemes that repeatedly need to compute some type of averaging is challenging. We have therefore implemented a flexible weighted vector adjustment SOM approach to group methylation profiles into clusters of similar global shape, despite potential local differences. These clusters then serve as methylation patterns, and can be correlated with time-series data on exon inclusion and intron retention.

To validate the method, we have applied our program to publicly available real-world data sets from eight tissues/cell lines. Our proof-of-concept results indicate the existence of tissue independent stable patterns which have a non-trivial correlation with AS events (exon inclusion and intron retention), which would hint at their role as regulators on an epigenetic level.

Further analysis on large-scale data sets is required to increase confidence in the patterns we found in the data that was available to us, and to establish further ones and study their respective roles. In fact, the patterns detected in our experiments are not the main contribution of our work, and should rather be treated as a proof-of-concept. The emphasis of our work is clearly on the method that has been developed. Nevertheless, as this software is explicitly designed with computational efficiency in mind, the required experiments can be easily performed in reasonable computational time. This is especially encouraging, since the amount of sequence methylation data is continuously increasing.

Due to the importance of AS events, considerable attention has been directed toward a better understanding and characterization of these events. For instance, recent studies have shown that AS events can be at least partially predicted from RNA-seq data (27–29). However, studying the effect of methylation patterns on AS in a tissue-independent manner computationally has, to the best of our knowledge, not yet been attempted, even though experiments hint at interesting non-trivial correlations (6–8,30,31). Thus, our method allows, for the first time, three kinds of studies: (i) researchers can use our software to detect novel methylation patterns and investigate their meaning; e.g. comparisons to non-alternatively spliced exons might help to unravel regulatory motifs; (ii) the software can be extended to query methylation data against a set of pre-computed profiles; (iii) the approach allows to study time-series data to see whether changes in AS events correlate with corresponding changes in methylation prototypes. For all these use cases, it is crucial that the software detects meaningful and stable prototypes. Our experiments indicate that this hinges upon the use of the DTW to provide a similarity measure for profiles that is relatively stable against common evolutionary events that leave the overall shape of the profile intact.

## AVAILABILITY

Our software is written in C/C++. Binaries and source code of the FSOM typing software for Windows32/64 and Linux64 are freely downloadable at http://sourceforge.net/projects/fsom/ (under the GPLv3 license). All tests have been conducted on a workstation with an Intel ® Core™ i7 CPU and 4 GB RAM.

The input data sets to the FSOM software are available at http://sourceforge.net/projects/fsom/files/Testing_data/. An excel sheet containing the details of the utilized AS events with inclusion information is available at http://sourceforge.net/projects/fsom/files/Exon_inclusion_data/.

## Supplementary Material

SUPPLEMENTARY DATA
