# Supplementary material for: A computational method for studying the relation between alternative splicing and DNA methylation
Source: Nucleic Acids Res. 2015 Sep 13;44(2):e19. doi: 10.1093/nar/gkv906 (PMC4737180; doi:10.1093/nar/gkv906)
Supplement: SUPPLEMENTARY DATA [file supp_44_2_e19__index.html]

A computational method for studying the relation between alternative splicing and DNA methylation — A computational method for studying the relation between alternative splicing and DNA methylation — SUPPLEMENTARY DATA 

# A computational method for studying the relation between alternative splicing and DNA methylation

## SUPPLEMENTARY DATA

- SUPPLEMENTARY DATA
