# Supplementary material for: A computational method for studying the relation between alternative splicing and DNA methylation
Source: Nucleic Acids Res. 2015 Sep 13;44(2):e19. doi: 10.1093/nar/gkv906 (PMC4737180; doi:10.1093/nar/gkv906)
Supplement: SUPPLEMENTARY DATA [file supp_gkv906_nar-01936-met-n-2015-File003.pptx]

## Slide 1
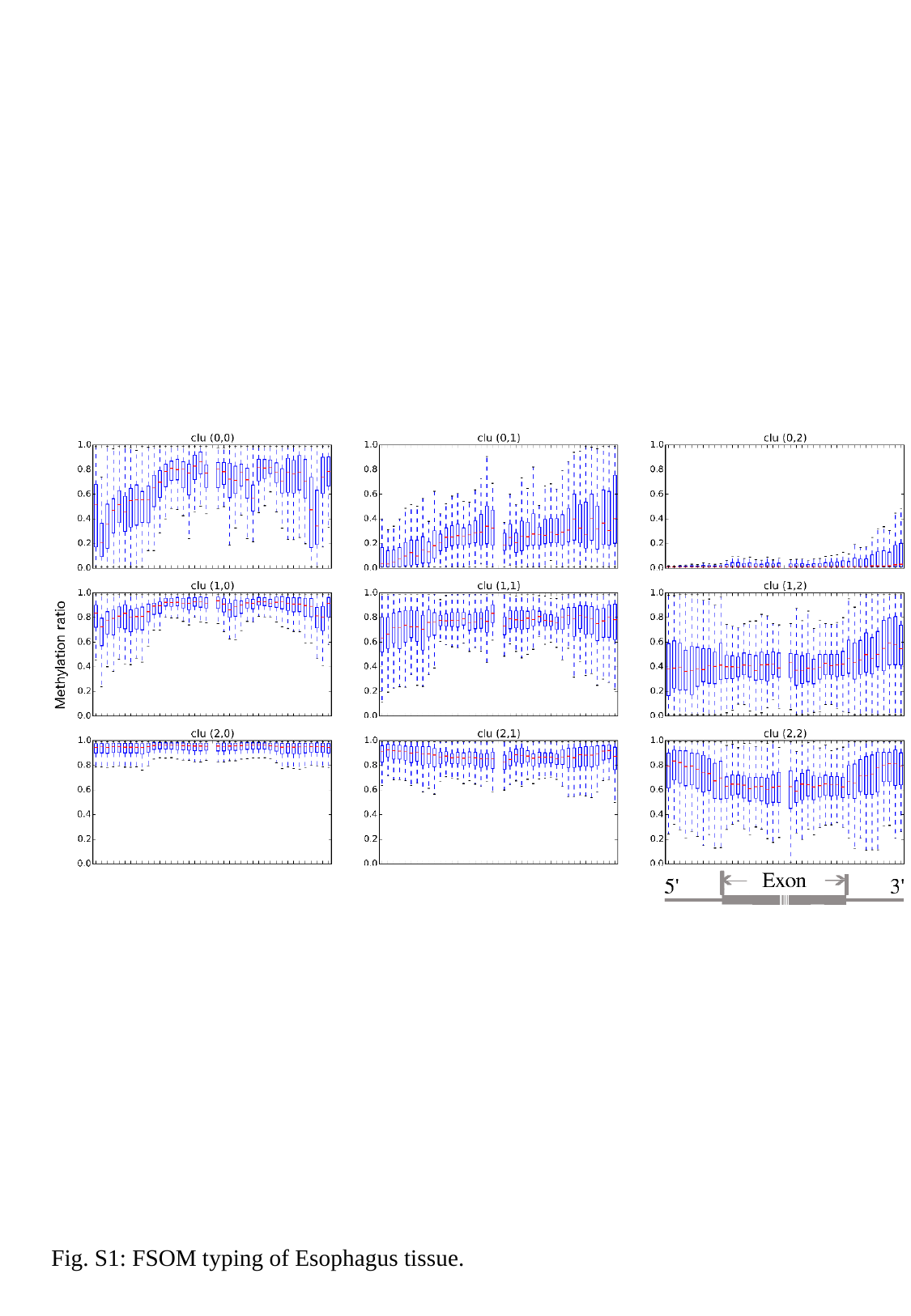

Fig. S1: FSOM typing of Esophagus tissue.

## Slide 2
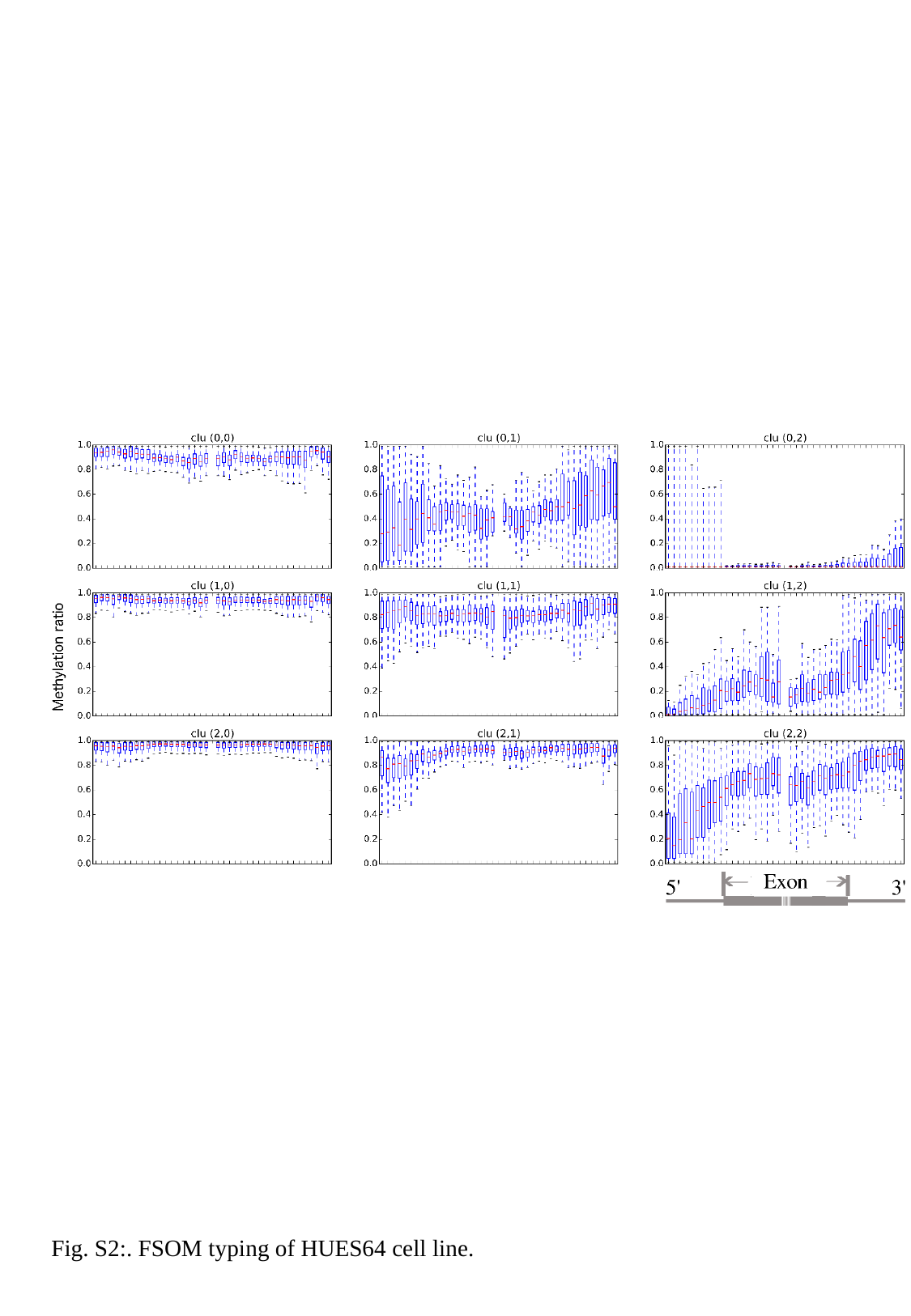

Fig. S2:. FSOM typing of HUES64 cell line.

## Slide 3
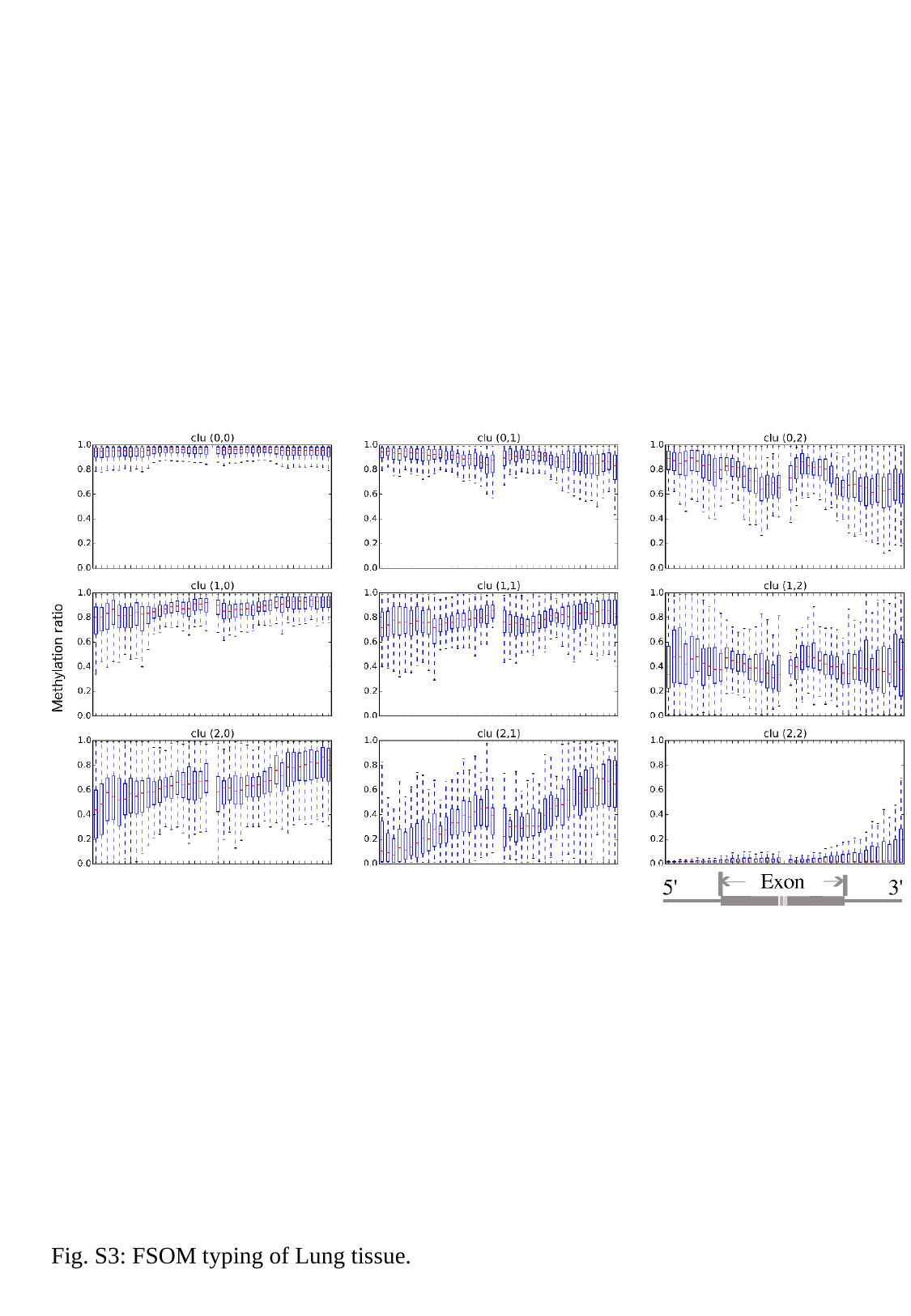

Fig. S3: FSOM typing of Lung tissue.

## Slide 4
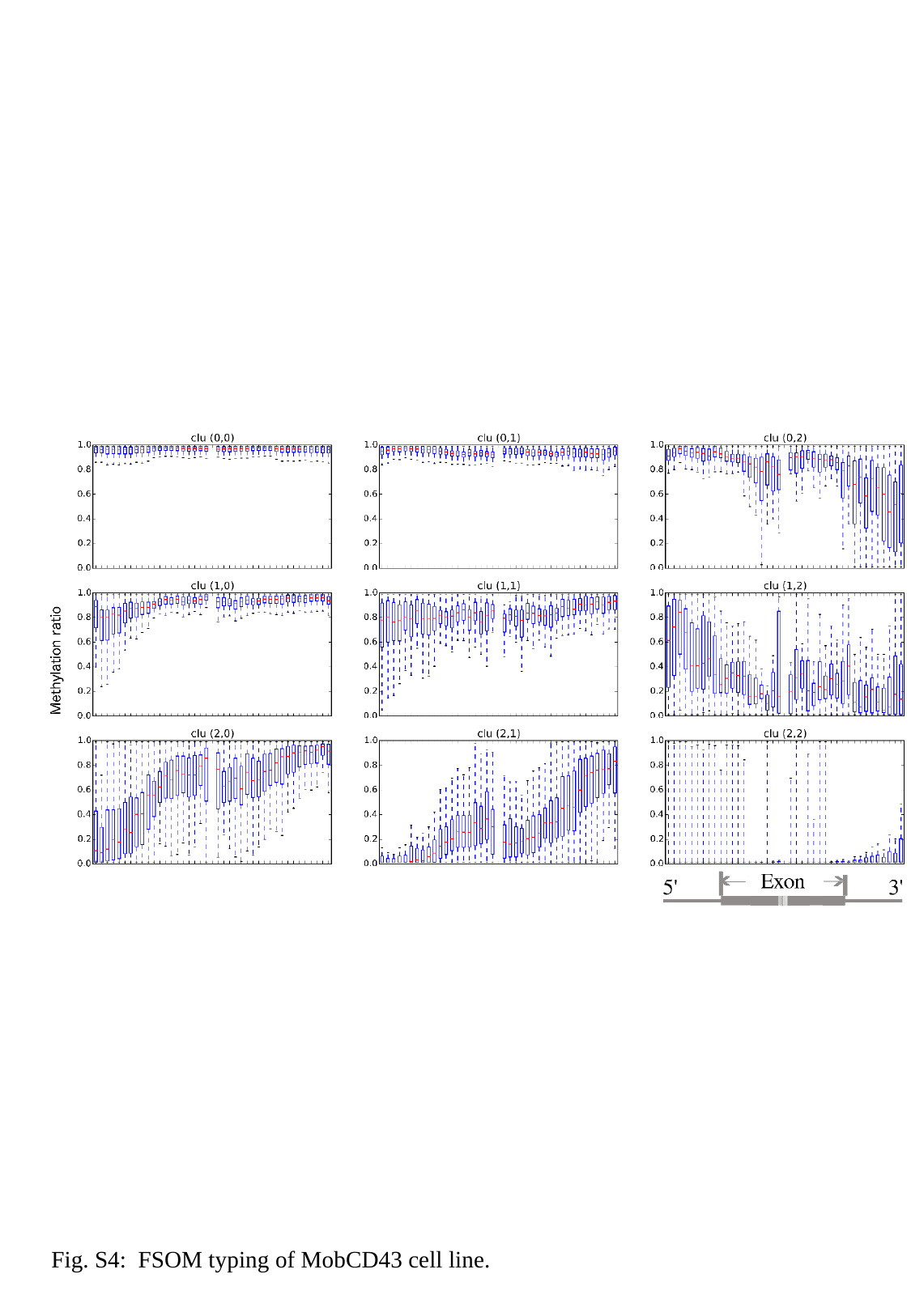

Fig. S4: FSOM typing of MobCD43 cell line.

## Slide 5
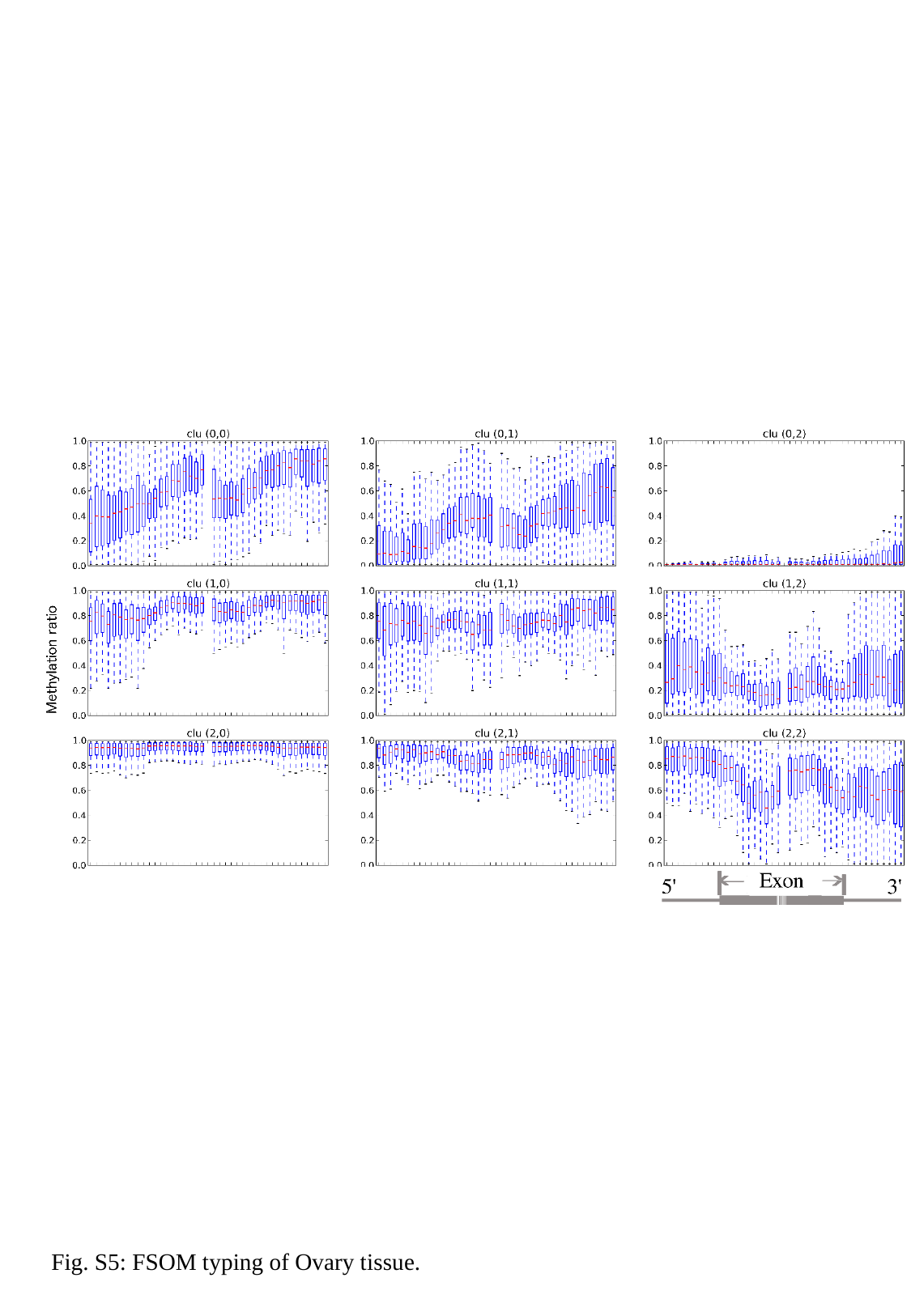

Fig. S5: FSOM typing of Ovary tissue.

## Slide 6
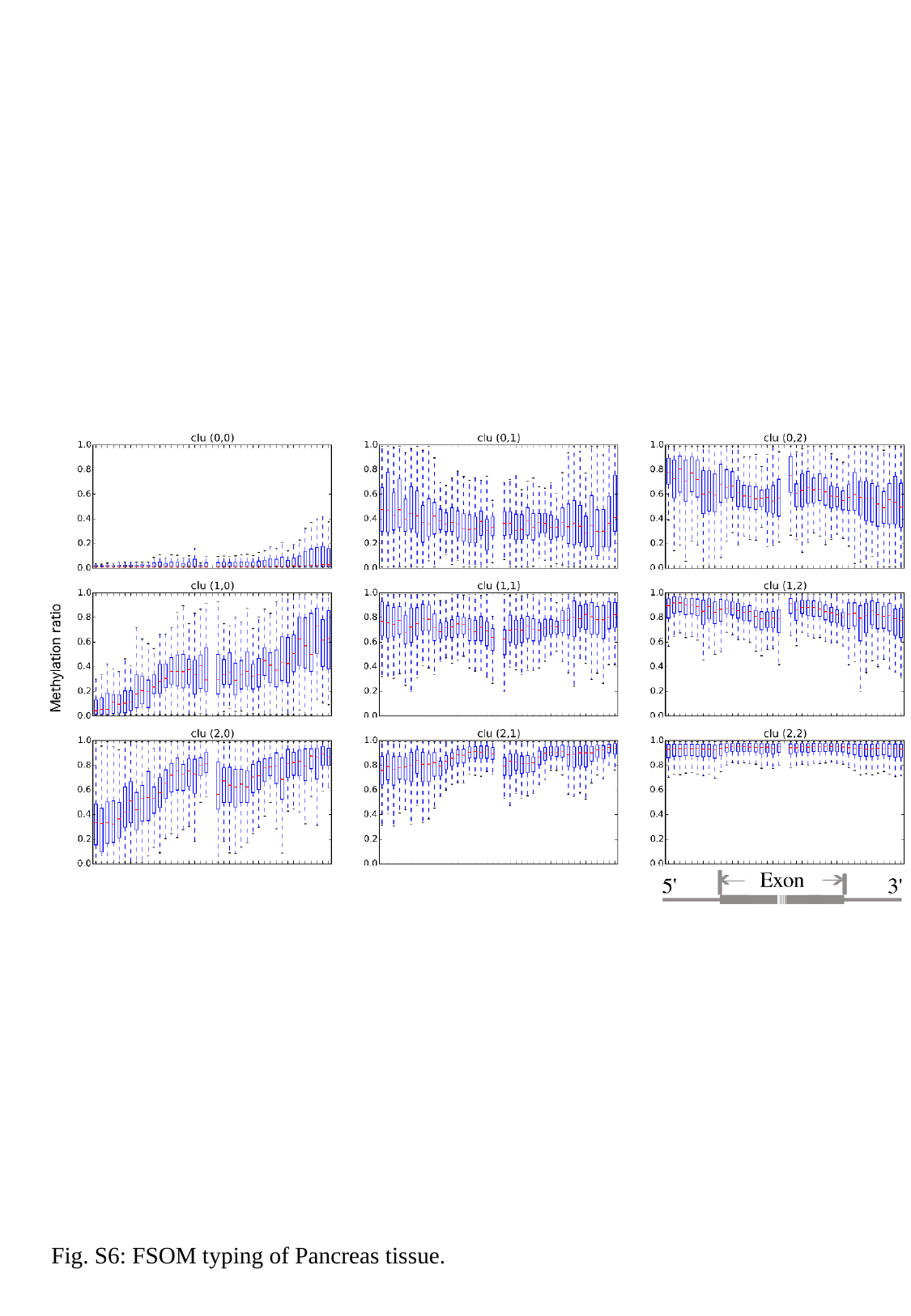

Fig. S6: FSOM typing of Pancreas tissue.

## Slide 7
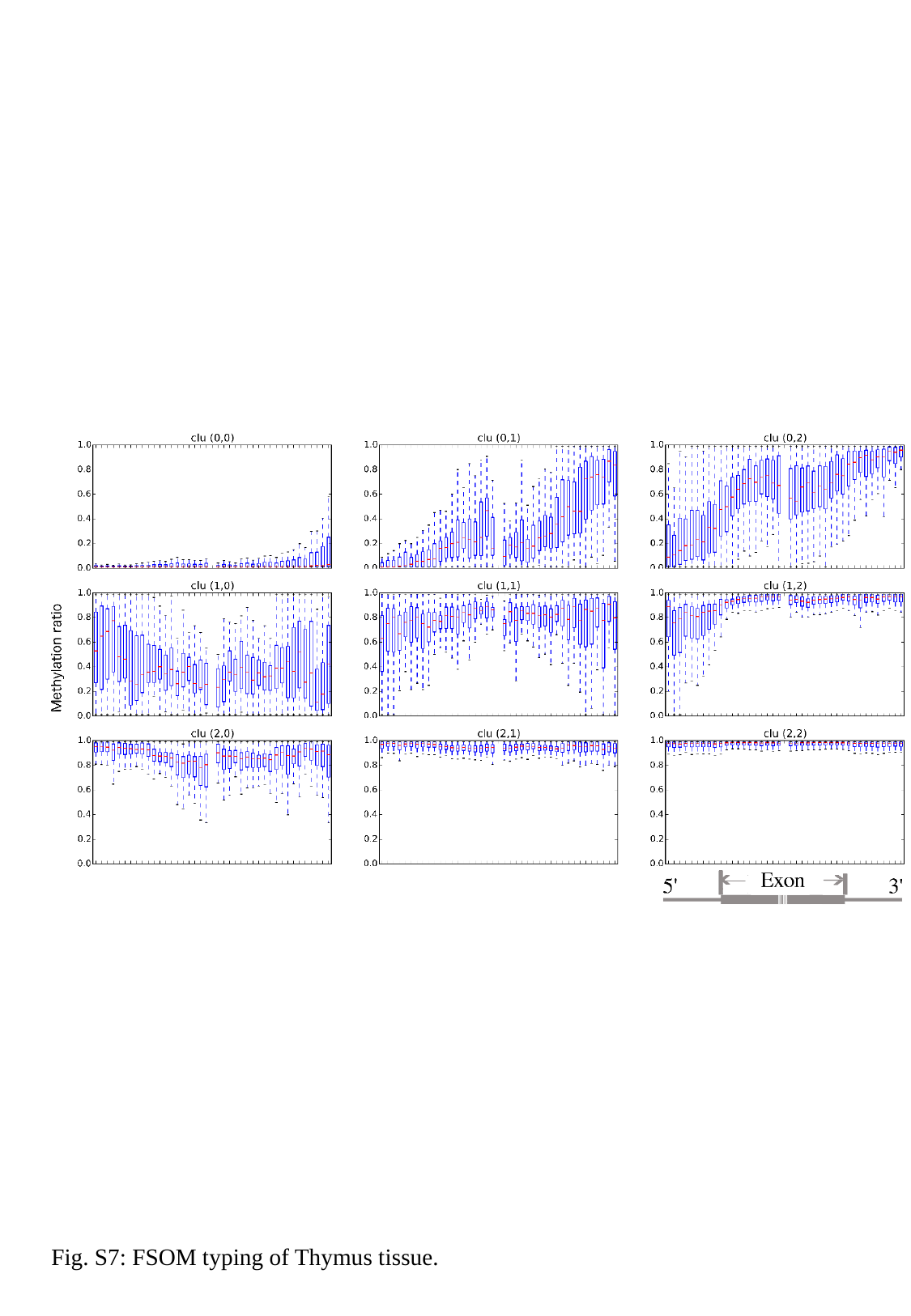

Fig. S7: FSOM typing of Thymus tissue.

## Slide 8
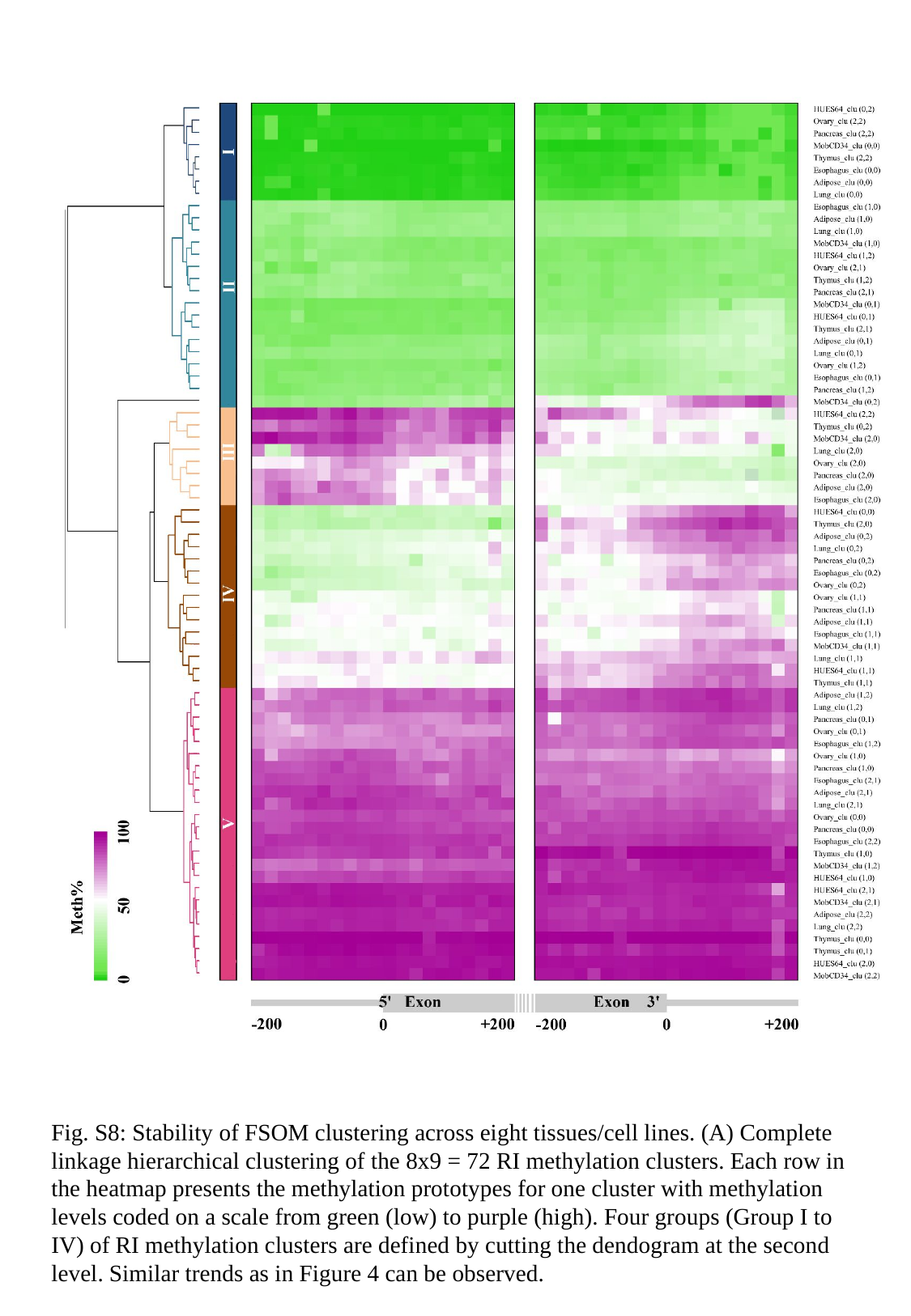

Fig. S8: Stability of FSOM clustering across eight tissues/cell lines. (A) Complete linkage hierarchical clustering of the 8x9 = 72 RI methylation clusters. Each row in the heatmap presents the methylation prototypes for one cluster with methylation levels coded on a scale from green (low) to purple (high). Four groups (Group I to IV) of RI methylation clusters are defined by cutting the dendogram at the second level. Similar trends as in Figure 4 can be observed.

## Slide 9
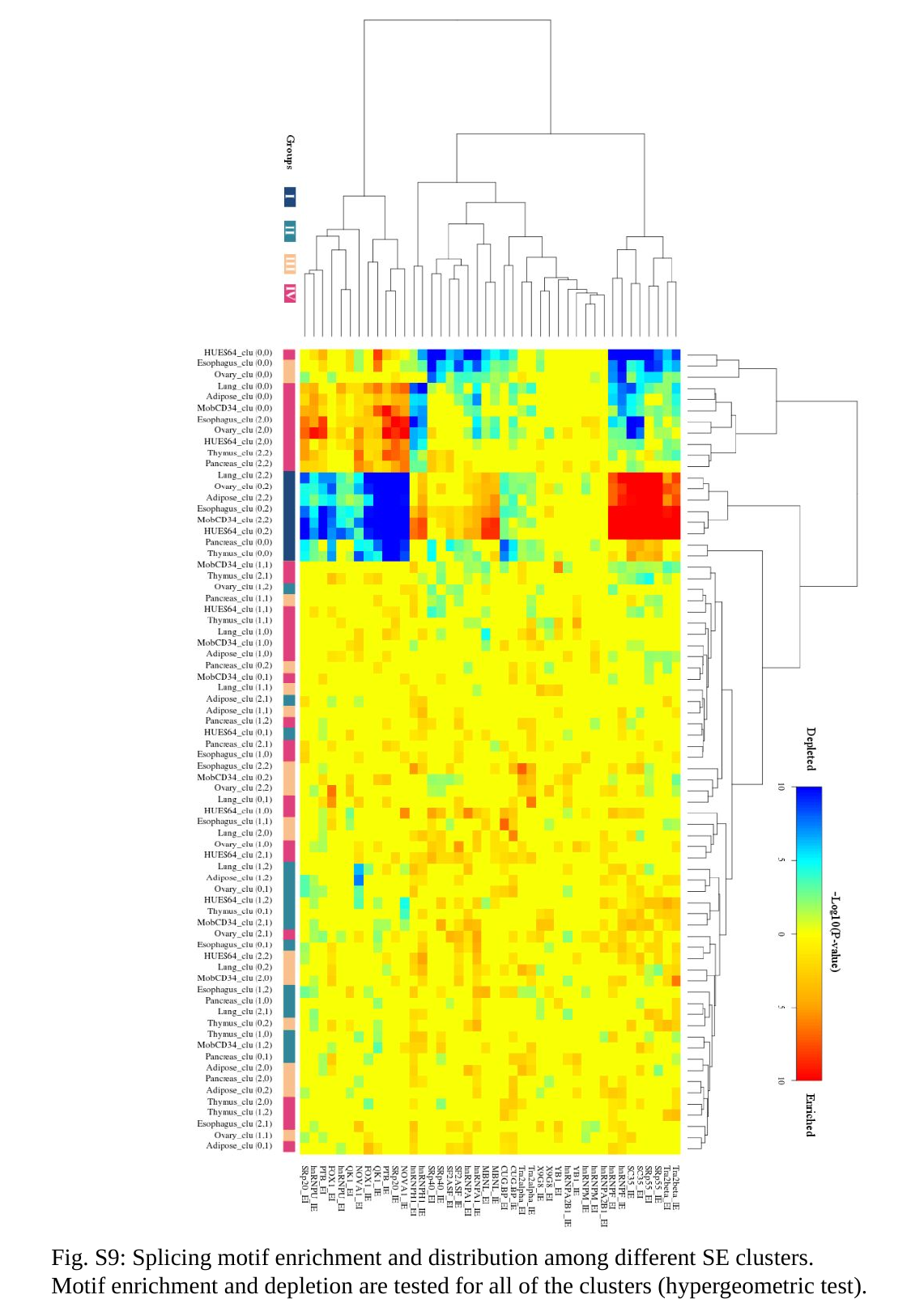

Fig. S9: Splicing motif enrichment and distribution among different SE clusters. Motif enrichment and depletion are tested for all of the clusters (hypergeometric test).

## Slide 10
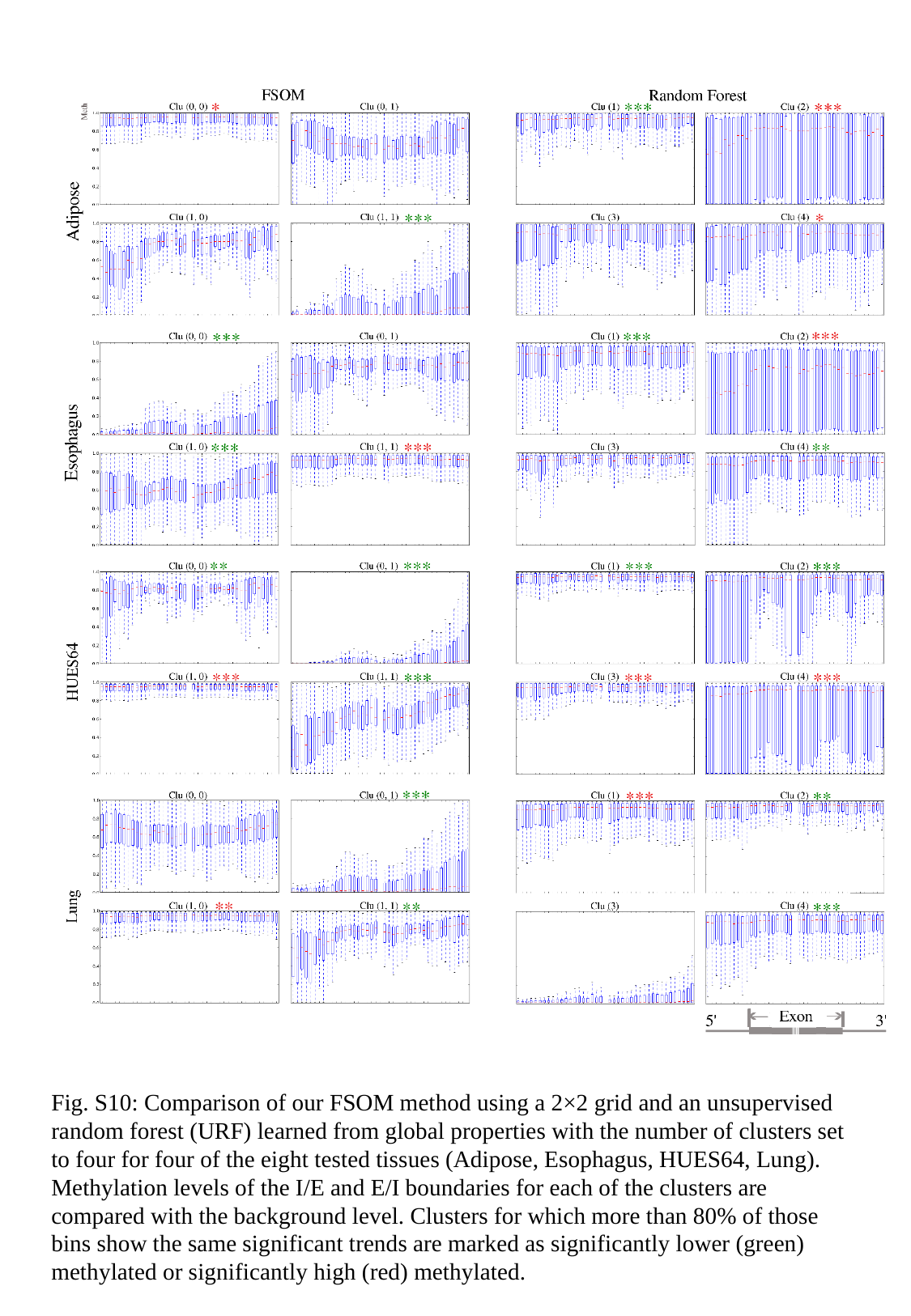

Fig. S10: Comparison of our FSOM method using a 2×2 grid and an unsupervised random forest (URF) learned from global properties with the number of clusters set to four for four of the eight tested tissues (Adipose, Esophagus, HUES64, Lung). Methylation levels of the I/E and E/I boundaries for each of the clusters are compared with the background level. Clusters for which more than 80% of those bins show the same significant trends are marked as significantly lower (green) methylated or significantly high (red) methylated.

## Slide 11
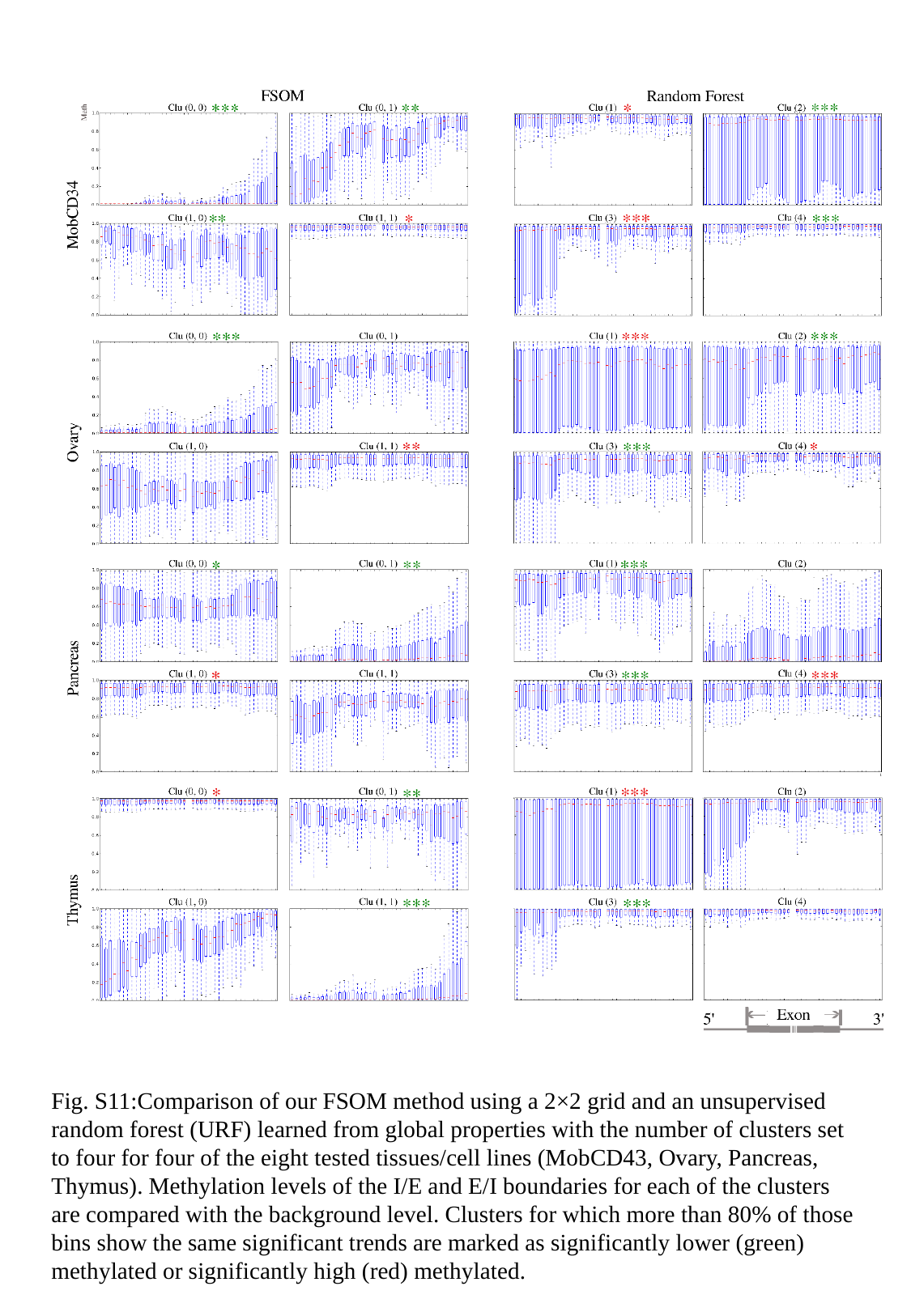

Fig. S11:Comparison of our FSOM method using a 2×2 grid and an unsupervised random forest (URF) learned from global properties with the number of clusters set to four for four of the eight tested tissues/cell lines (MobCD43, Ovary, Pancreas, Thymus). Methylation levels of the I/E and E/I boundaries for each of the clusters are compared with the background level. Clusters for which more than 80% of those bins show the same significant trends are marked as significantly lower (green) methylated or significantly high (red) methylated.
